# Swarming Aqua Sperm Micromotors for Active Bacterial Biofilms Removal in Confined Spaces

**DOI:** 10.1002/advs.202101301

**Published:** 2021-08-08

**Authors:** Carmen C. Mayorga‐Martinez, Jaroslav Zelenka, Jan Grmela, Hana Michalkova, Tomáš Ruml, Jan Mareš, Martin Pumera

**Affiliations:** ^1^ Center for Advanced Functional Nanorobots Department of Inorganic Chemistry University of Chemistry and Technology Prague Technicka 5, 166 28, Prague 6 Czech Republic; ^2^ Department of Biochemistry and Microbiology University of Chemistry and Technology Prague Technicka 5, 166 28, Prague 6 Czech Republic; ^3^ Department of Zoology Fisheries Hydrobiology and Apiculture Mendel University in Brno Zemedelska 1 Brno CZ‐61300 Czech Republic; ^4^ Department of Chemistry and Biochemistry Mendel University in Brno Zemedelska 1 Brno CZ‐613 00 Czech Republic; ^5^ Future Energy and Innovation Laboratory Central European Institute of Technology Brno University of Technology Purkynova 656/123 Brno CZ‐616 00 Czech Republic; ^6^ Center for Nanorobotics and Machine Intelligence Department of Food Technology Mendel University in Brno Zemedelska 1 Brno CZ‐613 00 Czech Republic; ^7^ Department of Chemical and Biomolecular Engineering Yonsei University 50 Yonsei‐ro, Seodaemun‐gu Seoul 03722 Korea; ^8^ Department of Medical Research China Medical University Hospital China Medical University No. 91 Hsueh‐Shih Road Taichung Taiwan

**Keywords:** active bacterial biofilms, Aqua Sperm micromotors, biobots, nanorobots, spermatozoa, spermbots

## Abstract

Microscale self‐propelled robots show great promise in the biomedical field and are the focus of many researchers. These tiny devices, which move and navigate by themselves, are typically based on inorganic microstructures that are not biodegradable and potentially toxic, often using toxic fuels or elaborate external energy sources, which limits their real‐world applications. One potential solution to these issues is to go back to nature. Here, the authors use high‐speed Aqua Sperm micromotors obtained from North African catfish (*Clarias gariepinus*, B. 1822) to destroy bacterial biofilm. These Aqua Sperm micromotors use water‐induced dynein ATPase catalyzed adenosine triphosphate (ATP) degradation as biocompatible fuel to trigger their fast speed and snake‐like undulatory locomotion that facilitate biofilm destruction in less than one minute. This efficient biofilm destruction is due to the ultra‐fast velocity as well as the head size of Aqua Sperm micromotors being similar to bacteria, which facilitates their entry to and navigation within the biofilm matrix. In addition, the authors demonstrate the real‐world application of Aqua Sperm micromotors by destroying biofilms that had colonized medical and laboratory tubing. The implemented system extends the biomedical application of Aqua Sperm micromotors to include hybrid robots for fertilization or cargo tasks.

## Graphical abstract

Ultra‐fast speed catfish Aqua Sperm micromotors that use water‐induced dynein ATPase catalyzed adenosine triphosphate (ATP) degradation as biocompatible fuel to trigger their snake‐like undulatory locomotion that facilitates biofilm destruction in less than one minute even in biofilms‐colonized medical and laboratory tubing.

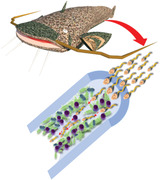

## 1 Introduction

Microrobotics is a relatively young field of research that has seen impressive growth in recent years. The field focuses on developing new propulsion methods, advanced materials with multiple functionalities, and sophisticated manufacturing methods that allow obtaining micro/nanorobots of different shapes and sizes.^[^ ^1^, ^2^, ^3^, ^4^, ^5^, ^6^, ^7^ ^]^ A plethora of useful applications for micro/nanorobots has been demonstrated in the fields of biomedicine, diagnosis, therapy, and environmental remediation.^[^ ^8^, ^9^, ^10^, ^11^, ^12^, ^13^ ^‐^ ^16^ ^]^ Many of these micro and nanomotors have been inspired by biological cell machinery that we find in nature as motile cells (bacteria, spermatozoa, microalgae) with cytoskeletal molecular motors, deoxyribonucleic acid (DNA)‐ or ribonucleic acid (RNA)‐processing enzyme motors, and ATPsynthase dual‐motor systems.^[^ ^17^, ^18^, ^19^ ^]^ There have been many efforts to utilize combinations of biological materials such as pollen, plant cells, and sperm cells, with spermatozoa being probably the most frequently used cell type.^[^ ^18^, ^20^, ^21^, ^22^ ^]^ The main advantages of using biological robots (biobots) are their biodegradability, on‐board power source, and biocompatibility because they generate energy from their surrounding biocompatible medium.^[^ ^18^ ^]^

Bacterial biofilms can easily colonize the surfaces of medical and laboratory devices, and are responsible for many nosocomial infections that cause recurrent and often intractable infectious diseases. Such bacterial biofilms can be more dangerous than planktonic bacteria. In fact, bacterial biofilms develop collective resistance to the immune response of the host and antibiotics, increasing the costs of treatments due to the use of expensive last‐generation antimicrobial drugs.^[^ ^23^, ^24^ ^]^ For this reason, the search for alternative treatments to remove bacterial biofilms is highly required and micro/nano scale motors have recently been used to disrupt bacterial biofilms.^[^ ^25^, ^26^, ^27^, ^28^ ^]^

**Scheme 1:**
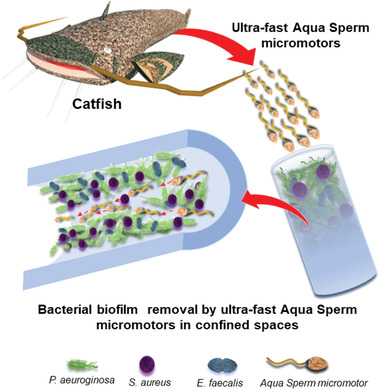
Schematic representation of ultra‐fast speed Aqua Sperm micromotors obtained from North African catfish destroying three species of bacterial biofilm colonized on medical and laboratory tubing.

Herein, we present a new concept, where we use Aqua Sperm micromotors to disturb bacterial biofilms. We use Aqua Sperm from North African catfish‐as microrobots with head sized ≈1.5 µm compared with, for example, bovine sperm of head sizes ≈10 µm.^[^ ^20^ ^]^ Their small size allows them to penetrate and disturb the biofilm matrix. Moreover, their ultra‐fast speed is efficient at destroying biofilms in a short time. Aqua Sperm micromotors have been used for destroying biofilms of prototypical pathogenic bacteria strains such as *Pseudomonas aeruginosa (P. aeruginosa), Staphylococcus aureus (S. aureus), and Enterococcus faecalis (E. faecalis)*.^[^ ^29^, ^30^, ^31^, ^32^ ^]^ The implementation of these Aqua Sperm micromotors resulted in the successful destruction of biofilm that colonized medical and laboratory tubing (**Scheme** **1**).

## 2 Results and discussion

**Figure 1:**
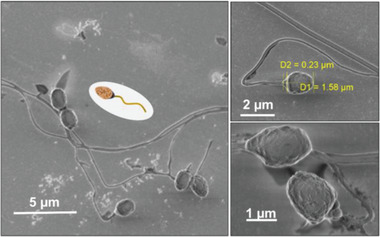
Scanning electron micrographs of catfish Aqua Sperm micromotors at different magnification

The study focused on using catfish‐derived Aqua Sperm micromotors to disrupt bacterial biofilm that colonized surfaces and tubing of medical and laboratory devices. These North African catfish Aqua Sperm micromotors move on average 9.5 bodylengths/s^33^ compared with 3 bodylengths/s for bovine sperm micromotors.^[^ ^21^ ^]^ As can be seen from the morphology of the catfish Aqua Sperm by scanning electron microscopy (SEM) (**Figure** **1**), they are about 10 times smaller than bovine sperm. Moreover, Aqua Sperm micromotors used in this work show homogeneous size and structure with a head size of ≈1.85 µm, a middle section of ≈0.23 µm, and a tail of more than 10 µm in length.

**Figure 2:**
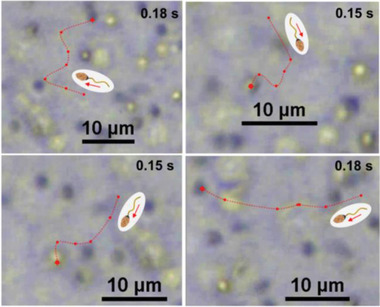
Tracked images of Aqua Sperm micromotor trajectories 15 s after initiating mobility by adding ultrapure water

To evaluate the mobility of the North African catfish Aqua Sperm micromotors, fresh Aqua Sperm micromotors were collected from the gonads of catfish previously euthanized and their motility was initiated by adding ultrapure water. Immediately after adding water, the Aqua Sperm micromotors started to move, reaching maximum speed after 15 s. **Figure** **2** shows the trajectories of different Aqua Sperm micromotors at their maximum speed. This evaluation demonstrated that the Aqua Sperm micromotors are viable, with an average velocity of 114 ± 11 µm/s, which is in good agreement with one reported previously.^[^ ^33^ ^]^ After 30 s, the Aqua Sperm micromotors slowed to a complete stop. Video S1 captures this whole process: Aqua Sperm micromotors before activation, during ultra‐fast movement, and deceleration until their complete stop. The mechanism of locomotion activation of Aqua Sperm micromotors is based on the decrease of seminal vesicle secretion viscosity in presence of water.^[^ ^34^ ^]^ Moreover, Aqua Sperm micromotors from African catfish have a naturally short lifetime probably due to an inadequate energy supply as fish sperms consume ATP very fast by dynein ATPase within the flagellar motile apparatus that drives sperm movement.^[^ ^35^ ^]^

Once the morphology and mobility of Aqua Sperm micromotors were evaluated, their interaction with the bacterial biofilm was studied. To perform these experiments, bacterial biofilms were prepared in Petri dishes for 24 h (see experimental details in the Materials and Methods section).

**Figure 3:**
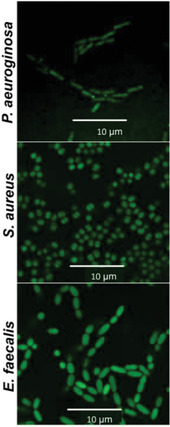
Confocal microscopy of *P. aeruginosa*, *S. aureus*, and *E. faecalis* stained with SYTO 9 DNA probe. Scale bars correspond to 10 µm.

**Figure 4:**
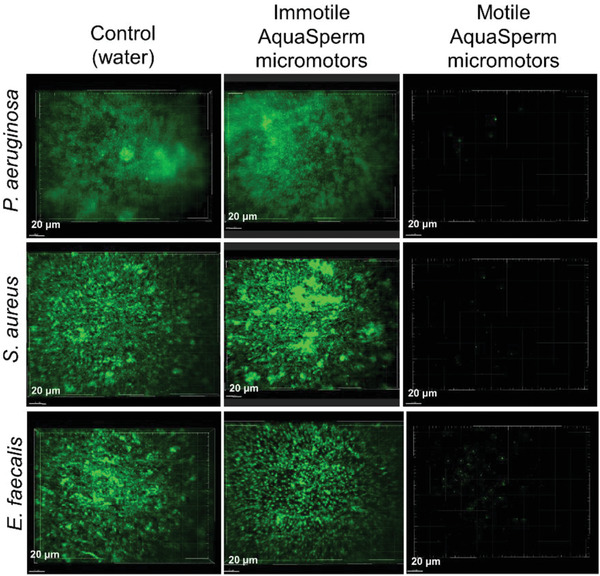
Confocal microscopy of bacterial biofilms of *P. aeruginosa, S. aureus*, and *E. faecalis* grown on Petri dishes for 24 h after treatment with water only (left panels), immotile (central panels), and motile (right panels) Aqua Sperm micromotors. Cells were stained with SYTO 9 DNA probe and images show maximal intensity projection of the confocal sections.

To evaluate the interaction between Aqua Sperm micromotors and the bacterial biofilms colonizing the Petri dishes, freshly collected Aqua Sperm micromotors were added and distributed homogeneously on the bacterial biofilm and ultra‐pure water was added. The ultra‐fast Aqua Sperm micromotors penetrated into the bacterial biofilm and disrupted it within 30 s. Videos S2, S3, and S4 show clearly how due to their snake‐like undulatory locomotion, the Aqua Sperm micromotors entered the biofilms and mechanically disrupted them completely in about 30 s. The ultra‐fast speed, short mobility time, and head size similar to bacteria, facilitated rapid biofilm disintegration because the Aqua Sperm micromotors were able to enter and navigate inside the biofilm matrix. When SEM images of Aqua Sperm micromotors (Figure 1) and confocal images of bacterial strains (**Figure** **3**) are compared, it can be observed that the head of Aqua Sperm micromotors (1.58 µm) is in the same size scale of *P. aeruginosa* (∼1.3 µm), *S. aureus* (∼1 µm), and *E. faecalis* (∼2.1 µm). In addition, the biodegradability of Aqua Sperm micromotors and their intrinsic bioenergy that can be released with water to trigger their motion confer great potential for biofilm treatment without creating any toxic products or requiring an external power source. Confocal microscopy showed the remaining biofilm of *P. aeruginosa*, *S. aureus*, and *E. faecalis* after treatment with Aqua Sperm micromotors (right panels of **Figure** **4**). The efficiency of the Aqua Sperm micromotors to disrupt biofilms of all bacterial strains can be clearly seen when compared with confocal microscopy of the remaining biofilm of *P. aeruginosa*, *S. aureus*, and *E. faecalis* after treatment with water (left panels of Figure 4).

A cultivation method was used to quantify the efficiency of bacterial biofilm disruption by ultra‐fast Aqua Sperm micromotors (see details in Materials and Methods section). For this aim, *P. aeruginosa*, *S. aureus*, and *E. faecalis* biofilms were grown for 24 h and 48 h in Petri dishes. The Aqua Sperm micromotor suspensions were homogeneously distributed on the biofilm surface and water was added. After 1 min, the Aqua Sperm micromotor suspension was removed and residual biofilms were harvested by ultrasound bath. To confirm that biofilms were efficiently harvested by ultrasound bath, the biofilms of *P. aeruginosa*, *S. aureus*, and *E. faecalis* treated by ultrasound bath were evaluated by confocal microscopy. Figure S1 shows clearly that few bacteria remain at the bottom of Petri dishes after ultrasound treatment.

**Figure 5:**
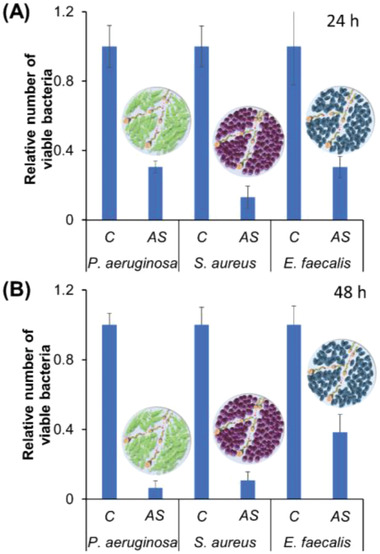
Relative number of viable bacteria in biofilms from *P. aeruginosa*, *S. aureus*, and *E. faecalis* grown for 24 h (**A**) and 48 h (**B**) before *C* (control) and after treatment with Aqua Sperm (AS) micromotors.

**Figure** **5** shows the relative number of viable bacteria in biofilms grown for 24 h (Figure 5A) and 48 h (Figure 5B) and treated with Aqua Sperm micromotors. These results were compared with biofilm where water was added instead Aqua Sperm micromotors. Strong disruption by Aqua Sperm micromotors was observed even in thicker biofilms grown for 48 h.

An additional control experiment using immotile Aqua Sperm micromotors was evaluated. For this aim, pure water was added to an Aqua Sperm cells solution. After one minute, this suspension was added to the bacterial biofilm grown for 24 h. No significant disruption by immotile Aqua Sperm micromotors was observed in all bacterial biofilms (see Figure S2 in Supporting Information). Moreover, confocal microscopy showed the remaining biofilm of *P. aeruginosa, S. aureus*, and *E. faecalis* after treatment with immotile Aqua Sperm micromotors (central panels of Figure 4). No effect of immotile Aqua Sperm micromotors was observed. This result corroborates that the efficiency of biofilm disruption by Aqua Sperm micromotors is due to their ultra‐fast speed.

Stability of the Aqua Sperm micromotors over time was evaluated. For this aim, the performance of Aqua Sperm micromotors collected after 2 and 9 days was compared during the treatment of *P. aeruginosa* biofilm. As can be seen in Video S5, the motors still moved after 9 days; however, when we evaluated them after 12 days, they had stopped moving completely (data not shown).

**Figure 6:**
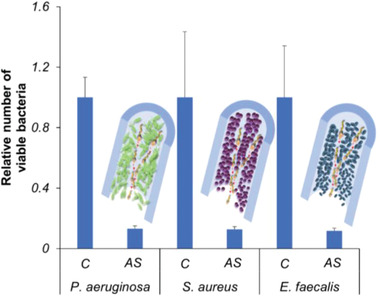
Relative number of viable bacteria in biofilms from *P. aeruginosa*, *S. aureus*, and *E. faecalis* grown for 24 h inside silicone tubing. Intact biofilm control: *C*; biofilm treated with Aqua Sperm micromotors: *AS*.

The tubing used with medical and laboratory devices is highly vulnerable to bacterial colonization, adhesion, and biofilm formation, which presents an important public health problem. In this way, hospital‐acquired infections are related to bacterial biofilms that grow in medical and laboratory tubing, which obligates their replacement and increases the cost and length of medical treatments as well as patient morbidity. For this aim, in this current study, silicone tubing was used as a model for medical and laboratory tubing. The tubing was pre‐colonized for 24 h with *P. aeruginosa*, *S. aureus*, and *E. faecalis* biofilms. Afterwards, the silicone tubing was treated with Aqua Sperm micromotors. Video S6 shows how ultra‐fast Aqua Sperm micromotors generated a strong flux inside the silicone tubing and disturbed the bacterial biofilm. In addition, the efficiency of bacterial biofilm disruption by ultra‐fast Aqua Sperm micromotors inside the silicone tubing was evaluated. After treatment with the Aqua Sperm micromotors, residual biofilms were collected from the tubing using an ultrasound bath and then quantified by cultivation method. **Figure** **6** compares the viability of different bacteria before and after they were treated with Aqua Sperm micromotors *S*. The obtained results show that the number of bacteria in the biofilms decreased by around 87% in all cases.

In general, the major problem with biofilm is its macromolecular network of extracellular matrix, which is highly mechanically resistant, dampens the flow in the tube, and may serve as a reservoir for unwanted molecules and germs. Destruction of this matrix by traditional methods, i.e., ethanol wash, bleach (NaClO) wash, and eventually ultrasound, is never complete and the biofilm is soon renewed from the remnants. Moreover, the enlisted methods may be incompatible with some kinds of plastic equipment due to mechanical fragility or chemical sensitivity. Traces of biomolecules remaining after the treatment with the Aqua Sperm micromotors are far less problematic than remnants of biofilm. Moreover, the catfish Aqua Sperm micromotors, unlike human sperm, does not form a fibrin precipitate, therefore its washout after the application is easy.

## 3 Conclusion

We have demonstrated that by using their undulatory locomotion, ultra‐fast Aqua Sperm micromotors can destroy efficiently different biofilms formed from *P. aeruginosa*, *S. aureus*, and *E. faecalis* bacterial strains. The efficient destruction of bacterial biofilms by Aqua Sperm micromotors in less than one minute may be attributed to their ultra‐fast speed and their undulatory locomotion as well as a head size similar to bacterial cells, allowing the Aqua Sperm micromotors to enter and navigate inside the biofilm matrix. Highly efficient bacterial biofilm destruction in pre‐colonized medical catheters treated with Aqua Sperm micromotors was also demonstrated. These results represent a promising and alternative solution to bacterial biofilm destruction using ultra‐fast, biodegradable, and biocompatible Aqua Sperm micromotors. This concept can be extended from tubing to other parts of medical devices and the non‐specific mechanical mode of action is a promise of universal application against other bacterial strains including mixed biofilms.

## 4 Materials and methods

### Bacterial biofilm preparation and destruction through Aqua Sperm micromotors

*P. aeruginosa*, *S. aureus*, and *E. faecalis* bacterial strains were obtained from the collection of opportunistic pathogens at the Department of Biochemistry and Microbiology, UCT Prague. Strains were propagated on Luria–Bertani (LB) agar at 37 °C and stored at 4 °C. Biofilms were prepared in LB medium inoculated with respective strains at optical density of 1 McFarland.

2 mL bacterial suspensions were added into Petri dishes (2 cm diameter) and incubated for 24 h and 48 h at 37 °C. After that, supernatants were gently removed and 50 µL of Aqua Sperm micromotors was homogeneously dropped on the surface of the biofilm and 500 µL of water added. After 1 min of Aqua Sperm micromotor action, the resulting supernatant with released bacterial cells was carefully removed and 500 µL of water was added. The remaining biofilm was removed from the surface of the Petri dish using an ultrasound bath.

To grow bacterial biofilm inside a medical catheter, a 0.5 cm‐long silicone tube with inside diameter of 4.8 mm was placed inside a plastic tube and 0.5 mL of bacterial suspension in LB was added and incubated for 24 h at 37 °C and then the supernatant was removed. After that, 50 µL of Aqua Sperm micromotors was placed inside the tube and 500 µL of water was added. After 1 min of treatment, the supernatant containing the released cells was removed and 500 µL of water was added to collect the remaining biofilm using an ultrasound bath.

Video showing the destruction of biofilm on the surface of the Petri dish and in the silicone tube was recorded using a Nikon inverted microscope with a 100X objective lens and Basler acA‐1920‐155 µm monochrome CMOS camera.

Confocal 3D imaging was performed with an Andor revolution xD system on an Olympus IX81 microscope operated with iQ3 software. Bacterial cells were stained with SYTO 9 DNA probe.

### Morphological characterization of Aqua Sperm micromotors

To obtain the SEM images, Aqua Sperm micromotors were fixed for 30 min in 1% glutaraldehyde at RT, washed with PBS, and then centrifuged at 600×g for 10 min. Aqua Sperm were dehydrated for 5 min in 40%, 50%, 60%, 70%, 80%, 90%, and for 10 min 100% ethanol; the final 100% alcohol was replaced twice. Final fixation was in hexamethyldisilazane for 30 min. SEM images were obtained using a scanning electron microscope, Tescan MAIA 3 (Tescan Ltd., Brno, Czech Republic) equipped with a field emission gun, cryogenic system PP3010 (Quorum Technologies Ltd., Sussex,UK).

### Broodstock handling and gametes collection

The broodstock was reared in controlled conditions at the Recirculating Aquaculture System (RAS), a registered experimental station of the Faculty of AgriSciences at Mendel University in Brno (breeding facility number CZ 62 060 420).

Gametes were obtained from mature males of a minimum weight of 1.5 kg. Males were chosen based on possession of pointed and hyperemic urogenital papilla.^[^ ^36^ ^]^ The sperm of North African catfish cannot be released by abdominal massage due to the presence of seminal vesicles.^[^ ^37^ ^]^ In this case, fish were percussively stunned and bled according to regulations and ethics.^[^ ^38^, ^39^ ^]^ Testes were removed by surgery from the fish belly, cleaned of blood, and cut into pieces using scissors. The sperm of each fish were released from cut gonads by squeezing through a 100‐micron mesh screen, collected into dry sterile containers, and stored at 4 °C.^[^ ^40^, ^41^ ^]^

### Motility evaluation of Aqua Sperm micromotors

To evaluate the motility of Aqua Sperm micromotors, 5 µL of their suspension was placed onto a glass slide and 50 µL of water was added and the video was recorded. Video sequences were recorded using a Nikon inverted microscope with a 100X objective lens and Basler acA‐1920‐155 µm monochrome CMOS camera; video processing was done using Nikon NIS‐Elements software.

### Viability evaluation of bacteria

Suspensions of bacteria released from biofilms by sonication were vortexed and serially diluted in water. Samples from different dilutions were transferred in 10 µL droplets on the surface of LB agar according to the Miles and Misra method, and incubated at 37°C overnight. Finally, colony‐forming units were calculated as spots with 4–40 colonies.

## Conflict of Interest

The authors declare no conflict of interest.

## Supporting information

Supporting Information

Supplemental Video 1

Supplemental Video 2

Supplemental Video 3

Supplemental Video 4

Supplemental Video 5

Supplemental Video 6
