# Supplementary material for: Swarming Aqua Sperm Micromotors for Active Bacterial Biofilms Removal in Confined Spaces
Source: Adv Sci (Weinh). 2021 Aug 8;8(19):2101301. doi: 10.1002/advs.202101301 (PMC8498868; doi:10.1002/advs.202101301)
Supplement: Supplementary file 1 — Supporting Information [file ADVS-8-2101301-s003.pdf]

© 2021 Wiley-VCH GmbH

# Supporting Information

for *Adv. Sci.,* DOI: 10.1002/advs.202101301

# Swarming Aqua Sperm Micromotors for Active Bacterial Biofilms Removal in Confined Spaces

Carmen C. Mayorga-Martinez, Jaroslav Zelenka, Jan Grmela, Hana Michalkova, Tomas Ruml, Jan Mareš, Martin Pumera*

# Supporting Information

# Swarming Aqua Sperm Micromotors for Active Bacterial Biofilms Removal in Confined Spaces

Carmen C. Mayorga-Martinez, Jaroslav Zelenka, Jan Grmela, Hana Michalkova, Tomas Ruml, Jan Mareš, Martin Pumera*

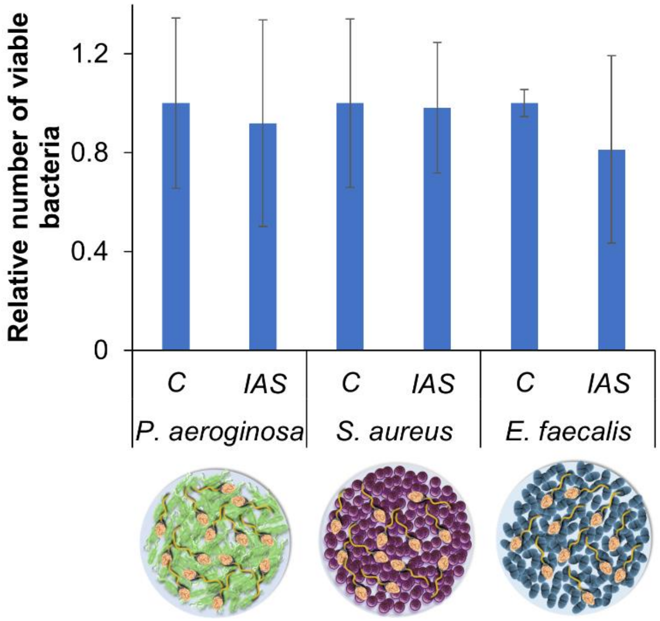


**Figure S1**. Relative number of viable bacteria in biofilms from *P. aeruginosa*, *S. aureus*, and *E. faecalis* grown for 24 h before (*C*) and after treatment with immotile AquaSperm micromotors (*IAS*).

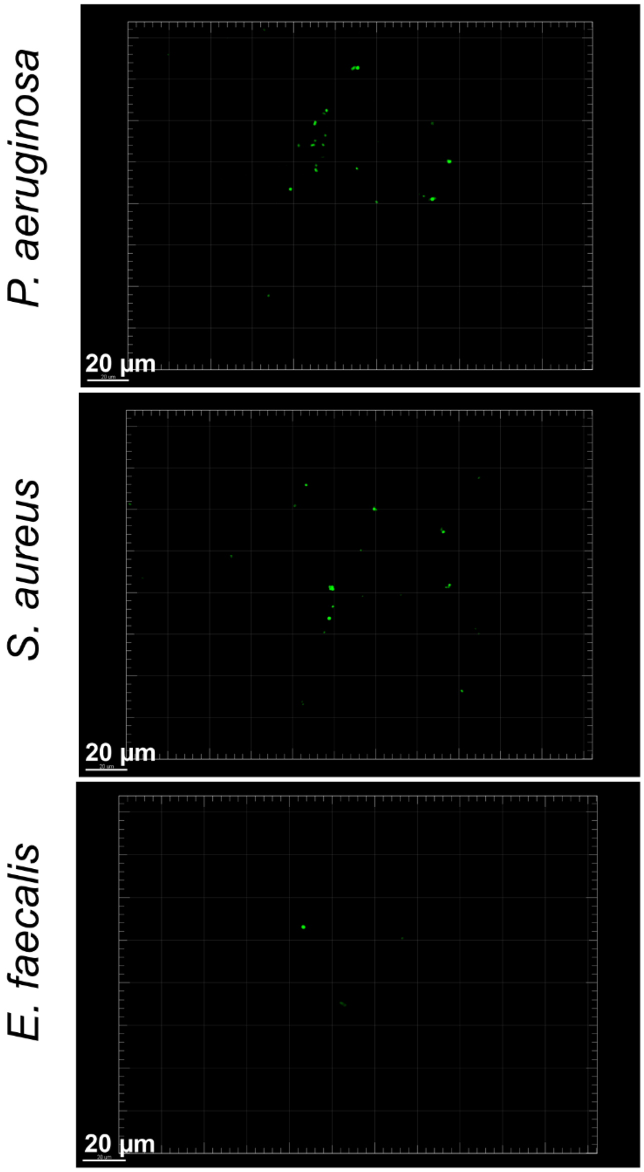


**Figure S2**. Confocal microscopy of bacterial biofilms of *P. aeruginosa*, *S. aureus*, and *E. faecalis* grown on Petri dishes for 24 h after treatment with ultrasound only. Cells were stained with SYTO 9 probe and images show maximal intensity projection of the confocal sections.
